# An Unusual Culprit: Staphylococcus capitis Native Mitral Valve Endocarditis Following Scalp Surgery

**DOI:** 10.7759/cureus.95301

**Published:** 2025-10-24

**Authors:** Silvia Sacramento Azenha, Bhavna Murugesh, Bhiramah Rammanohar, Sani Aliyu

**Affiliations:** 1 Department of Infectious Diseases, Addenbrooke's Hospital, Cambridge University Hospitals NHS Foundation Trust, Cambridge, GBR; 2 Clinical School of Medicine, University of Cambridge, Cambridge, GBR

**Keywords:** coagulase-negative staphylococcus, echocardiography, infection microbiology, infective discitis, native mitral valve endocarditis, staphylococcus capitis

## Abstract

*Staphylococcus capitis* is a coagulase-negative *Staphylococcus* (CoNS) that colonizes the skin, particularly the scalp, and is often dismissed as a blood culture contaminant in the absence of prosthetic material. Reports of invasive adult infections, particularly native valve endocarditis (NVE), are rare. We describe the case of a man in his 50s, previously healthy and with no known cardiac pathology, who presented with a three-week history of night sweats, myalgia, chest discomfort, and severe neck pain. Clinical examination revealed a pansystolic murmur, and five sets of blood cultures were positive for *S. capitis.* Echocardiography demonstrated large mitral valve vegetations with leaflet perforation and severe regurgitation. MRI revealed cervical discitis. The clinical course was complicated by antibiotic-associated hepatic cholestasis, rash, and thrombocytopenia, necessitating multiple antimicrobial switches. Ultimately, the patient underwent successful mechanical mitral valve replacement and completed a prolonged antibiotic course. Although *S. capitis* is frequently dismissed as a contaminant, this case highlights its potential to cause severe invasive disease, including NVE and discitis, even in immunocompetent adults without prosthetic devices or comorbidities. The unusual flucloxacillin susceptibility of the isolate further underscores the variability of antimicrobial resistance patterns in *S. capitis*. Clinicians should maintain a high index of suspicion when* S. capitis* is repeatedly isolated from blood cultures, and prompt echocardiography is essential to prevent diagnostic delay and adverse outcomes.

## Introduction

*Staphylococcus capitis* is a coagulase-negative Gram-positive cocci that is part of the normal flora of human skin, mainly the scalp and chin, and is usually regarded as a contaminant in the context of a positive blood culture in the absence of prosthetic material [1]. Recently, however, both adult and neonatal *S. capitis* infections have gained attention. Outbreaks of *S. capitis* in neonatal intensive care units have highlighted its subacute presentation in late-onset sepsis and significant antimicrobial resistance to most drug classes [2]. Limited literature describes adult infections with *S. capitis*, especially cases of native valve endocarditis (NVE). One case report has described prosthetic valve *S. capitis* endocarditis after a scalp laceration [3]. Of the 12 cases of *S. capitis* NVE cases reported, only one occurred in a patient with native valves in the absence of predisposing comorbidities or cardiac pathology [4]. In this case report, we present a rare case of *S. capitis*-associated NVE without established risk factors, following a scalp procedure. 

## Case presentation

History and examination 

A man in his 50s, previously well, presented with a three-week history of night sweats, myalgia, chest discomfort, and progressive neck pain. He denied recent dental procedures or intravenous drug use. Ten months earlier, he had undergone excision of a benign scalp lesion. On examination, he was afebrile with stable vital signs. Cardiovascular examination revealed a grade III/VI pansystolic murmur radiating to the axilla, with no clinical signs of heart failure. Neurological examination was unremarkable.

Investigations 

On admission, the C-reactive protein was 122 mg/L, the white cell count was 10.4 × 10⁹/L, and neutrophils were 7.9 × 10⁹/L. Three sets of blood cultures grew Gram-positive cocci in clusters, subsequently identified as *S. capitis *by matrix-assisted laser desorption/ionization-time-of-flight mass spectrometry (MALDI-TOF), a standard diagnostic technique freely used in clinical microbiology laboratories. Blood culture timeline and results are outlined in Table 1, and the antibiotic susceptibility testing of the first blood culture is detailed in Table 2. The isolate demonstrated cefoxitin susceptibility, indicating methicillin sensitivity and, by inference, susceptibility to flucloxacillin as per European Committee on Antimicrobial Susceptibility Testing (EUCAST) guidance [5]. Disc susceptibility interpretation was similar across all five blood culture sets.

**Table 1:** Blood culture timeline and results.

Timeline from admission	Blood culture results
Day 1	*Staphylococcus capitis* 2/2 bottles
Day 1	*Staphylococcus capitis* 2/2 bottles
Day 1	*Staphylococcus capitis *2/2 bottles
Day 2	*Staphylococcus capitis* 1/2 bottles
Day 3	*Staphylococcus capitis* 1/2 bottles

**Table 2:** Antibiotic susceptibility testing of first blood culture. EUCAST: European Committee on Antimicrobial Susceptibility Testing; E-test: epsilometer test; MIC: minimum inhibitory concentration

Antibiotic	Method	Value	Interpretation as per EUCAST
Cefoxitin (flucloxacillin inferred from this)	disc	32 mm	Susceptible
Erythromycin	disc	26 mm	Susceptible
Tetracycline	disc	32 mm	Susceptible
Rifampicin	disc	38 mm	Susceptible
Gentamicin	disc	26 mm	Susceptible
Linezolid	disc	28 mm	Susceptible
Clindamycin	disc	23 mm	Susceptible
Co-trimoxazole	disc	33 mm	Susceptible
Ciprofloxacin	disc	28 mm	Susceptible to increased exposure
Vancomycin	E-test MIC	1.0 mg/L	Susceptible
Teicoplanin	E-test MIC	0.125mg/l	Susceptible
Daptomycin	E-test MIC	0.5 mg/L	Susceptible

**Figure 1:**
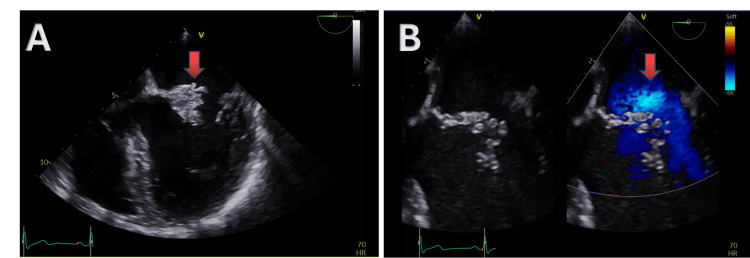
Transoesophageal echocardiogram (TOE) demonstrating mitral valve involvement. A: large vegetation on the anterior mitral valve leaflet (red arrow); B: color Doppler showing severe mitral regurgitation (red arrow).

**Figure 2:**
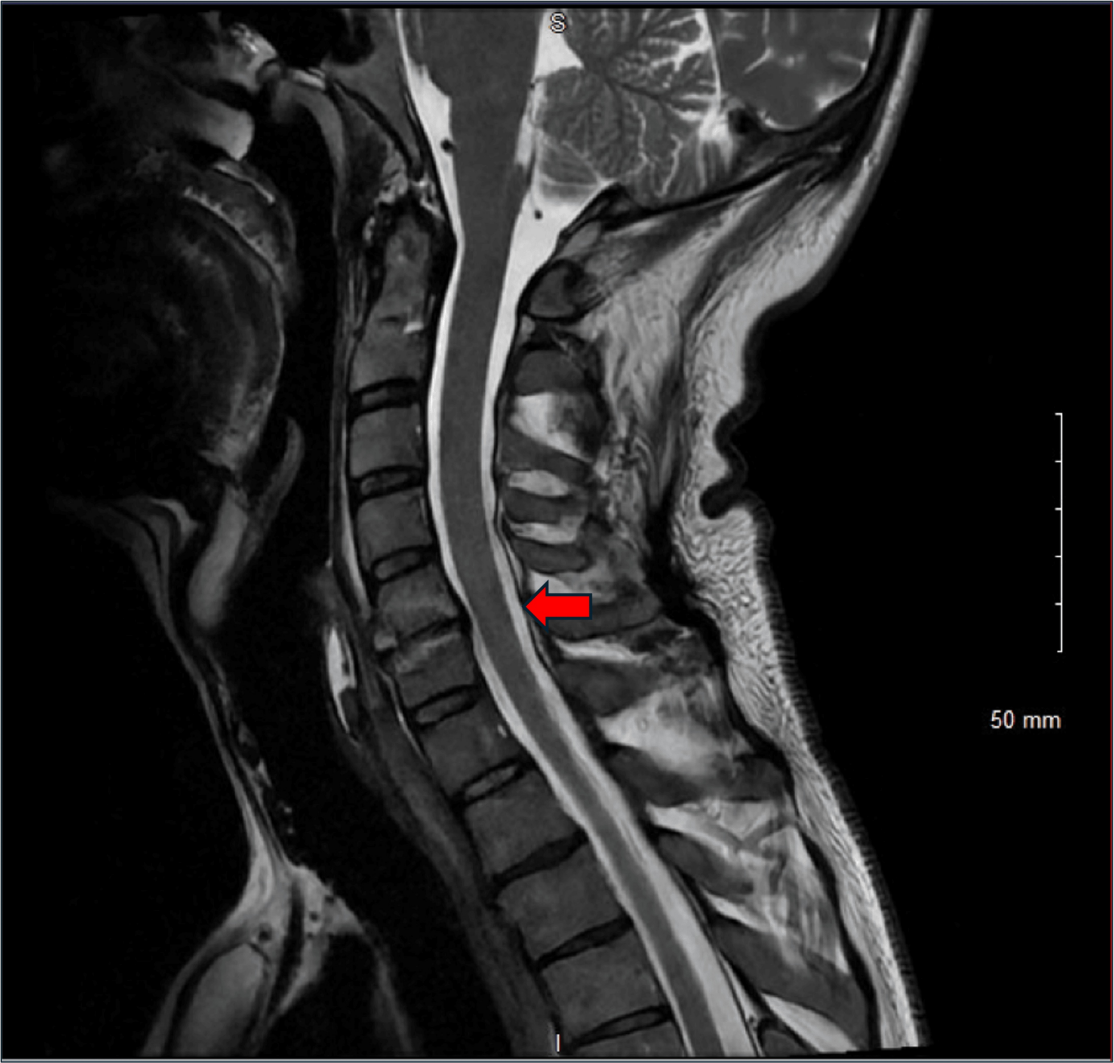
Magnetic resonance imaging (MRI) of the cervical spine showing infective discitis at C5–C6 (red arrow).

Transoesophageal echocardiography performed on day 10 demonstrated mitral valve vegetations involving both the anterior and posterior leaflets. The anterior leaflet had a 2.1 × 2.2 cm vegetation with a 1.2 cm mobile filament, while the posterior leaflet was thickened with a 0.7 × 1.2 cm mobile vegetation. There was also evidence of anterior leaflet perforation with severe mitral valve regurgitation (Figure 1). In view of persistent neck pain, an MRI of the whole spine was performed on day 15 to evaluate for possible spinal involvement, which revealed findings consistent with cervical infective discitis at the C5-C6 level (Figure 2). Imaging findings are summarized in Table 3. According to the 2023 Duke-International Society for Cardiovascular Infectious Diseases (ISCVID) criteria, this case meets the definition of definite infective endocarditis, based on repeated isolation of a nontypical organism (*Staphylococcus* *capitis*) from multiple blood culture sets and echocardiographic evidence of mitral valve vegetations with leaflet perforation and severe regurgitation [6].

**Table 3:** Imaging findings during admission and follow-up. MV: mitral valve; CT-TAP: CT thorax, abdomen, and pelvis; EF: ejection fraction

Timeline from admission	Imaging	Findings
Day 5	Transthoracic Echocardiogram (TTE)	1.1cm x 1.0cm vegetation on the anterior MV leaflet with regurgitation
Day 10	Transoesophageal Echocardiogram (TOE)	Mitral valve vegetation with anterior leaflet perforation and severe regurgitation
Day 13	CT-TAP and CT-Head	No evidence of septic embolisation
Day 15	MRI Spine	Cervical infective discitis (C5-C6)
Day 103	Transthoracic Echocardiogram (TTE)	EF- 45% and a well-seated mechanical mitral valve replacement with no significant stenosis or regurgitation

Treatment course 

Initial empirical antibiotics (amoxicillin and ceftriaxone) were changed to vancomycin after MALDI-TOF identification, and subsequently to flucloxacillin after susceptibility testing. On day five, clearance of the organism in blood cultures was achieved. Flucloxacillin was discontinued due to obstructive cholestasis, with liver function tests showing alanine aminotransferase (ALT) 267 IU/L (reference range 40-50 IU/L) and alkaline phosphatase (ALP) 202 IU/L (reference range 30-130 IU/L). The patient was then switched back to vancomycin (minimum inhibitory concentration (MIC) 1.0 mg/L). However, this was complicated by a blanching rash, neutropenia, and thrombocytopenia, which were thought to be induced by vancomycin. Therapy was subsequently changed to intravenous daptomycin. Following this, an improvement in cell counts was observed (Table 4).

**Table 4:** Hematological parameters before and after switch from vancomycin to daptomycin. L: low; IV: intravenous

Haematological parameters	On IV vancomycin	After switching to IV daptomycin
White blood cell (WBC) count (3.9 - 10.2 10*9/L )	0.6 (L)	4.4
Red blood cell (RBC) count (4.30 - 5.75 10*12/L )	4.61	4.34
Hemoglobin (Hb) (135 - 172 g/L )	139	128 (L)
Platelet (PLT) count (150 - 370 10*9/L )	63 (L)	152
Hematocrit (Hct) (0.395 - 0.505 L/L )	0.404	0.394 (L)
Lymphocyte count (1.10 - 4.50 10*9/L )	0.42 (L)	2.28
Basophil count (0.00 - 0.20 10*9/L )	0.01	0.02
Eosinophil count (0.00 - 0.50 10*9/L )	0.02	0.22
Monocyte count (0.10 - 0.90 10*9/L )	0.01 (L)	0.31
Neutrophil count (1.50 - 7.70 10*9/L )	0.12 (L)	1.47 (L)

Outcome 

The patient was transferred to the regional cardiothoracic center on day 32 of admission and underwent mechanical mitral valve replacement the following day. He completed 47 days of intravenous daptomycin and was discharged on oral linezolid for seven days. Enriched heart valve cultures were sent and did not demonstrate any growth. At the two-month follow-up, echocardiography showed improved ejection fraction (from 30-35% to 45%) and a well-seated mechanical mitral valve replacement with no significant stenosis or regurgitation. A gradual improvement in neck pain was also noted. 

## Discussion

Repeated isolation of coagulase-negative *Staphylococcus* (CoNS) from blood cultures can represent a range of possibilities, including true infective endocarditis, catheter- or device-related infection, transient bacteremia, or simple contamination [1]. Differentiating these is essential, as CoNS are frequent skin commensals. In this patient, the absence of intravascular or prosthetic material, together with consistent clinical findings and echocardiographic evidence, supported the diagnosis of true NVE. In a large multicenter cohort study, coagulase-negative s*taphylococci* accounted for approximately 12% of native valve infective endocarditis cases [7].

The European Society of Cardiology (ESC), American Heart Association (AHA), and National Institute for Health and Care Excellence (NICE) guidelines place emphasis on common pathogens associated with infective endocarditis, such as *Staphylococcus aureus* [8-10]. Coagulase-negative *staphylococci*, known for their biofilm formation and indolent course, are primarily discussed in the context of prosthetic valve endocarditis, with less focus on native valve infections. They can also infect other indwelling devices such as pacemaker leads, vascular grafts, central lines, and orthopedic implants, where biofilm formation facilitates persistent infection. Because these organisms are often associated with prosthetic material, *S. capitis *isolated from blood cultures may be mistakenly regarded as a contaminant in patients without such devices, leading to potential diagnostic delays [1,2].

This case highlights the significance of considering *S. capitis* as a cause of infective endocarditis affecting native heart valves, with potential complications including valve perforation and secondary infections, such as discitis. Systemic complications described in the literature also include vertebral osteomyelitis, meningitis, prosthetic joint infections, prosthetic valve endocarditis, and late-onset neonatal sepsis. Hematogenous spread has been implicated in device-related infections, intravascular catheter sepsis, and endovascular involvement such as pacemaker lead and prosthetic graft infections [1,2,4]. These reports demonstrate the organism’s potential for persistence and dissemination through biofilm formation on foreign materials, although this was not the underlying mechanism in our patient, who had no prosthetic devices.

A key learning point was the initial delay in echocardiography, as the organism was initially disregarded as a contaminant. This reflects the evolving concept of infective endocarditis as a time-sensitive diagnosis, where early echocardiography may help avoid diagnostic delay and prevent advanced complications [11].

Interestingly, the *S. capitis* isolate in this case was susceptible to flucloxacillin, which is uncommon given the high prevalence of methicillin resistance among coagulase-negative *staphylococci* due to *mecA* gene carriage [5].

Despite flucloxacillin being appropriate in this case, treatment was complicated by drug-induced obstructive cholestasis followed by a rash and thrombocytopenia, probably due to vancomycin. This underscores the need for vigilance and monitoring for drug toxicity when treating infective endocarditis.

As far as we know, previously reported cases of *S. capitis* NVE were associated with comorbidities or underlying valvular abnormalities such as mitral valve prolapse or bicuspid aortic valve [4]. In contrast, our patient had no known past medical history or structural heart disease, with the recent scalp procedure being the only identifiable risk factor and likely portal of entry for *S. capitis* bacteremia [12]. Colley et al. similarly reported *S. capitis* endocarditis following a scalp procedure; however, that case occurred in a patient with a prosthetic valve [3].

There was no growth in the enriched culture from the valve tissue, likely due to the patient having already received more than four weeks of antibiotics by that time. Although a broad-range 16S rDNA polymerase chain reaction (PCR) on the native valve would have been valuable in confirming *S. capitis* as the causative organism [13], the repeated isolation of the same organism in five initial blood cultures provided strong evidence in our case.

## Conclusions

*Staphylococcus capitis *can cause severe native mitral valve endocarditis with complications such as discitis, even in immunocompetent adults without comorbidities. Prior skin procedures may represent a risk factor. Clinicians should be cautious not to dismiss *S. capitis* as a contaminant when it is repeatedly isolated in blood cultures in patients with clinical features of infective endocarditis.

Prompt echocardiography is recommended to avoid diagnostic delays. This case underscores the need for vigilance in interpreting the significance of coagulase-negative staphylococci when repeatedly isolated in blood cultures, the variability of antimicrobial susceptibility, and the importance of supporting investigations to enable early and tailored therapy.
